# The impact of artificial light at night on nocturnal insects: A review and synthesis

**DOI:** 10.1002/ece3.4557

**Published:** 2018-10-23

**Authors:** Avalon C. S. Owens, Sara M. Lewis

**Affiliations:** ^1^ Department of Biology Tufts University Medford Massachusetts

**Keywords:** artificial light at night, bioluminescence, fireflies, light pollution, nocturnal insects, visual ecology

## Abstract

In recent decades, advances in lighting technology have precipitated exponential increases in night sky brightness worldwide, raising concerns in the scientific community about the impact of artificial light at night (ALAN) on crepuscular and nocturnal biodiversity. Long‐term records show that insect abundance has declined significantly over this time, with worrying implications for terrestrial ecosystems. The majority of investigations into the vulnerability of nocturnal insects to artificial light have focused on the flight‐to‐light behavior exhibited by select insect families. However, ALAN can affect insects in other ways as well. This review proposes five categories of ALAN impact on nocturnal insects, highlighting past research and identifying key knowledge gaps. We conclude with a summary of relevant literature on bioluminescent fireflies, which emphasizes the unique vulnerability of terrestrial light‐based communication systems to artificial illumination. Comprehensive understanding of the ecological impacts of ALAN on diverse nocturnal insect taxa will enable researchers to seek out methods whereby fireflies, moths, and other essential members of the nocturnal ecosystem can coexist with humans on an increasingly urbanized planet.

## 1 INTRODUCTION

Localized illumination of nocturnal landscapes by anthropogenic sources of light such as street lamps, path lights, and vehicle headlights, hereafter referred to collectively as artificial light at night (ALAN), is likely to disrupt populations of crepuscular and nocturnal animal species present in affected habitats (Davies & Smyth, 2018; Gaston & Holt, 2018; Navara & Nelson, 2007; Rich & Longcore, 2006). Recent global surveys of night sky brightness have concluded that 23% of land surfaces between 75°N and 60°S, including 88% of Europe and 47% of the United States, experience nightly “light pollution” in the form of an increase in night sky brightness at least 8% above the natural level (Falchi et al., 2016) and that this artificial illumination is gradually invading biodiversity hot spots (Guetté, Godet, Juigner, & Robin, 2018). A meta‐analysis of night sky brightness over time has found regional increases ranging from 0% to 20% per year, averaging 6% (Hölker, Moss, et al., 2010). Such increases are expected to match the pace of urban expansion (Elvidge et al., 2014). In the past two decades, however, some regions have experienced net decreases in night sky brightness, which may be due to environmental and economic incentives to reduce ALAN, as well as to technological innovations such as shielded lights that mitigate light trespass by restricting artificial illumination to desired areas (Bennie, Davies, Duffy, Inger, & Gaston, 2014, but see Kyba et al., 2017).

**Figure 1:**
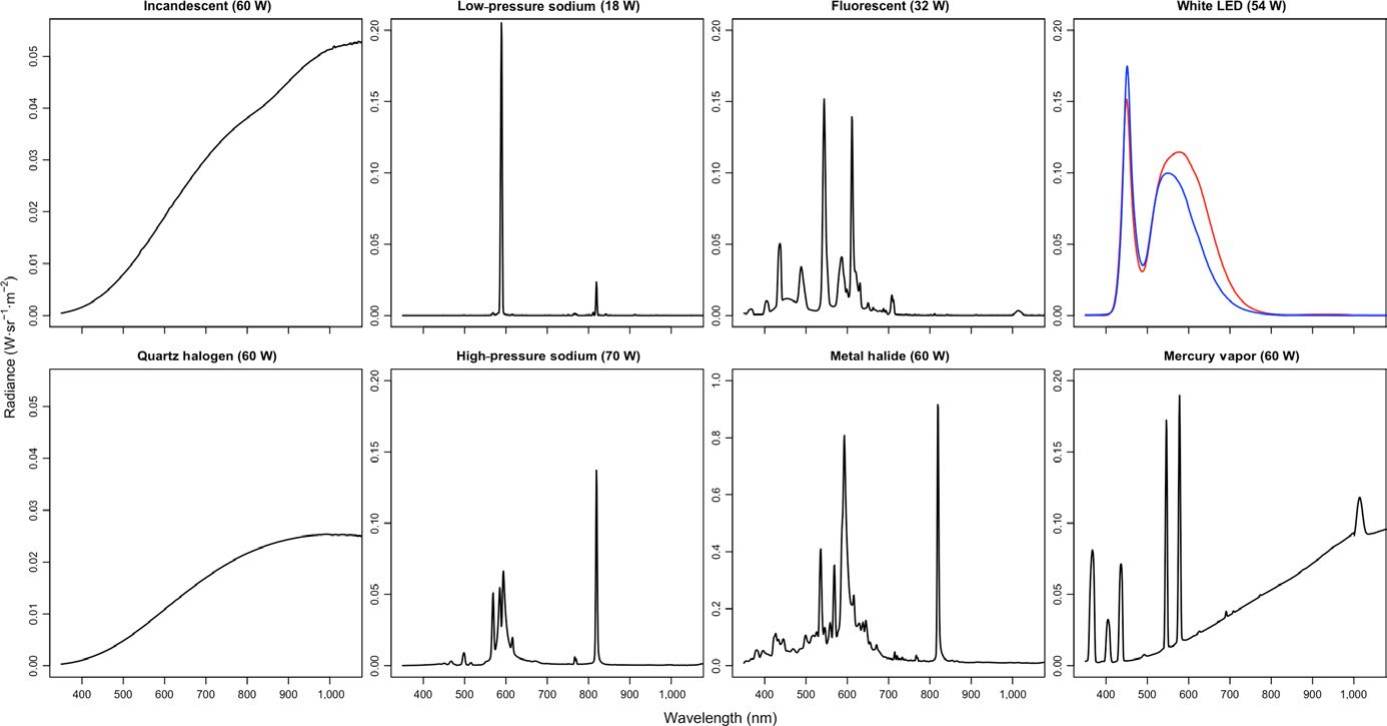
Spectral emission of different ALAN types. ALAN sources, such as incandescent and halogen bulbs, and mercury vapor lamps emit large amounts of energy as infrared radiation (heat); mercury vapor and metal halide lamps also emit a non‐negligible amount of UV radiation. Low‐pressure sodium lamps and LEDs are comparatively efficient ALAN sources, both capable of emitting nearly monochromatic visible light (see text). Neutral (red) and cool (blue) temperature white LEDs have been plotted on the same graph for comparison. Modified from Elvidge, Keith, Tuttle, and Baugh (2010) with permission

The ecological effects of ALAN depend on both its intensity and its spectral composition (as well as its flicker rate; see Inger, Bennie, Davies, & Gaston, 2014 for a review). Historically, ALAN sources have mainly comprised low‐ and high‐pressure sodium lamps, which emit characteristic emission spectra concentrated in the yellow‐to‐orange region of the visible spectrum, in addition to “whiter” broad‐spectrum mercury vapor lamps, which also emit a large amount of UV radiation (Figure 1). Most recently, environmental and economic concerns have galvanized movements to replace these traditional, energy‐intensive light sources with energy‐efficient alternatives, primarily LEDs (Davies, Bennie, Inger, & Gaston, 2013; Gaston, Davies, Bennie, & Hopkins, 2012). LEDs can emit monochromatic light of any desired wavelength within or adjacent to the visible spectrum; white light of a given color temperature is produced by coating blue LEDs in a phosphor material that absorbs a percentage of this light and re‐emits it in longer wavelengths (Krames et al., 2007). As a result of this process, most commercial white LED street lamps emit more of their light in the blue region of the visible spectrum than do other ALAN types (Figure 1). Exposure to blue light at night is known to cause insomnia and increased disease risk in humans (American Medical Association, 2016). However, the effects of this widespread spectral shift on other organisms have only recently attracted the attention of researchers (Davies et al., 2017; Donners et al., 2018; Gaston, Visser, & Hölker, 2015; Justice & Justice, 2016; Lewanzik & Voigt, 2017; Longcore et al., 2015, 2018 ; Pawson & Bader, 2014; Plummer, Hale, O'Callaghan, Sadler, & Siriwardena, 2016; Somers‐Yeates, Hodgson, McGregor, Spalding, & Ffrench‐Constant, 2013; Spoelstra et al., 2015; van Grunsven et al., 2014; van Langevelde, Grunsven, Veenendaal, & Fijen, 2017; Wakefield, Broyles, Stone, Jones, & Harris, 2016).

Worldwide, around 30% of vertebrates and more than 60% of invertebrates are nocturnal (Hölker, Wolter, Perkin, & Tockner, 2010). These species are undoubtedly most vulnerable to artificial illumination; they also tend to be underrepresented in the scientific literature. To bring greater attention to the ecological consequences of human activity in an often ignored temporal niche, herein we review known individual‐level impacts of ALAN on nocturnal animals, organized into the following five categories: temporal and spatial disorientation, attraction, desensitization, and recognition. Temporal disorientation covers both alterations in circadian clocks and photoperiodism as well as the partitioning of activity between day and night (sensu Gaston, Bennie, Davies, & Hopkins, 2013). To distinguish between discrete effects of ALAN on nocturnal insect taxa, visual perception (sensu Gaston et al., 2013) has been subdivided into the categories of recognition and desensitization; similarly, spatial orientation (sensu Gaston et al., 2013) has been subdivided into spatial disorientation and attraction. As the behavioral responses of individuals determine how ALAN will affect populations, and then entire ecological communities, we believe that this level of assessment is illuminating.

Recent studies have suggested that insect diversity and abundance are both undergoing a rapid decline (Hallmann et al., 2017). Insects are essential components of all terrestrial food webs, and any losses in insect biomass are likely to have widespread ecological ramifications. ALAN affects insects in unique ways related to their body size and visual system. We have therefore chosen to focus our review around nocturnal and crepuscular insects, although our classification may be applicable to other organisms as well. Previous reviews cover the effects of ALAN on plants (Bennie, Davies, Cruse, & Gaston, 2016), butterflies (Seymoure, 2018), and stream and riparian ecosystems (Perkin et al., 2011), as well as the effects of artificial light regimes on pest insects (Johansen, Vänninen, Pinto, Nissinen, & Shipp, 2011) and poultry (Van Nuffel, Bujis, & Delezie, 2015); for general reviews of the impacts of ALAN on all species, see Rich and Longcore (2006) and Gaston et al. (2013).

Fireflies (Coleoptera: Lampyridae), click beetles (Coleoptera: Elateridae), and glowworms (Diptera: Keroplatidae) are among the most charismatic of all nocturnal insects, and their unique light‐based communication system may make them especially vulnerable to artificial illumination. We therefore conclude with a review and synthesis of existing studies concerning the impact of ALAN on bioluminescent insects, followed by recommendations for future research.

## 2 INSECT VISION

As background for understanding the ecological effects of ALAN, some familiarity with how light is measured, and how nocturnal insects perceive light, is necessary. These topics are briefly introduced below; for a more in‐depth primer, see Cronin et al. (2014) and Honkanen, Immonen, Salmela, Heimonen, and Weckström (2017).

**Figure 2:**
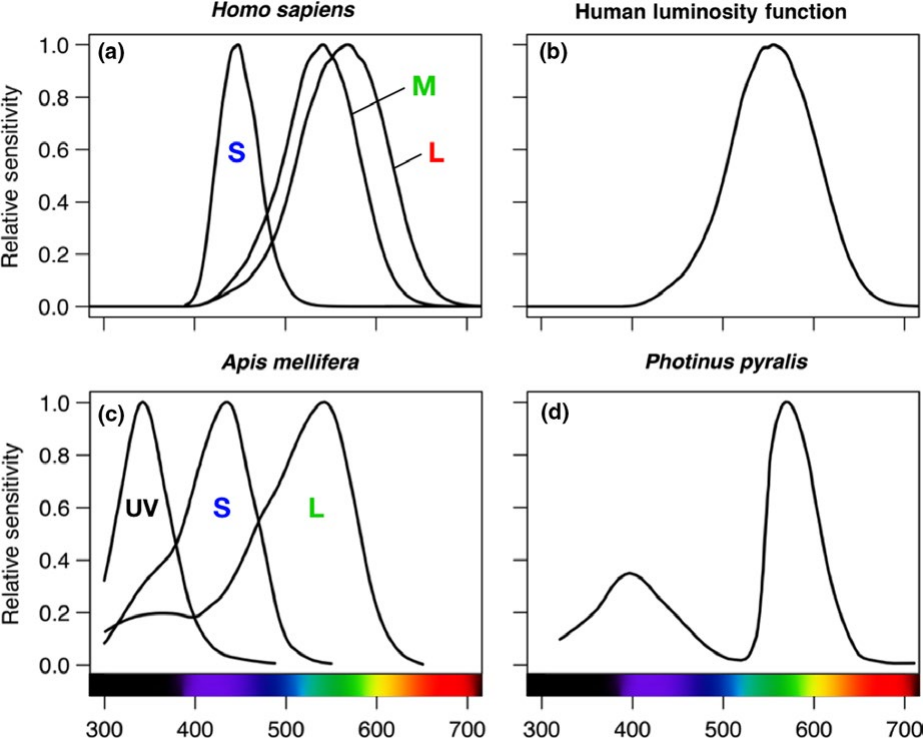
Spectral sensitivity in humans and insects. (a) Spectral sensitivities of blue (S, short‐wavelength), green (M, mid‐wavelength), and red (L, long‐wavelength) sensitive photoreceptors in humans presented as 10° cone fundamentals, calculated from Stiles and Burch (1959) color matching functions (Stockman & Sharpe, 2000; www.cvrl.org/). Humans are insensitive to UV wavelengths (<390 nm), perceive visible light wavelengths (390–700 nm) as color, and perceive infrared wavelengths (>700 nm) as heat. (b) Human luminosity function, used to predict relative “brightness” as perceived by humans. (c) Spectral sensitivity of the photoreceptors of honeybee workers, thought to have retained the trichromatic color vision of ancestral insects (Briscoe & Chittka, 2001; modified from Peitsch et al., 1992). (d) Electroretinography of a male Big Dipper firefly *Photinus pyralis* suggests that this species may have lost its short‐wavelength sensitive opsin (modified from Lall, Chapman, Ovid Trouth, & Holloway, 1980)

Light behaves both as a wave and as a particle (or photon). Wavelength is measured in nanometers (nm), with wavelengths between 400 and 700 nm corresponding to colors visible to humans. Intensity (particle density) is measured in units of counts per unit area (photons cm^−2^ s^−1^), with more photons corresponding to greater intensity. Brightness, a subjective perception of intensity, is influenced by individual spectral sensitivity, which is generally viewed as fixed for a given species. In trichromatic humans, who have blue‐, green‐, and red‐sensitive photoreceptors, luminance sensitivity peaks around 555 nm under photopic (well‐lit) conditions (Figure 2a,b). Photon counts can be weighted by this function to obtain measurements of brightness as perceived by the human eye, given in units of *lumens* (lm). Light emitted from a point source can be standardized to *candelas*, or lumens per solid angle (cd = lm/sr), and light incident on a flat surface is converted to *lux*, or lumens per area (lux = lm/m^2^). Although lux is widely used by engineers and policymakers, and is easiest to measure, it poorly approximates brightness as it is perceived by non‐human animals (Longcore & Rich, 2004).

Ancestral insects likely possessed three types of photoreceptors or opsins (Briscoe & Chittka, 2001; Figure 2c): one sensitive to ultraviolet wavelengths (UV, 300–400 nm), another to short wavelengths (blue, 400–480 nm), and a third to long wavelengths (green to amber, 480–600 nm). Subsequent evolution into different ecological niches and light environments then selected for variation in spectral sensitivity (Endler, 1993; Lall, Seliger, Biggley, & Lloyd, 1980; Théry, Pincebourde, & Feer, 2008). In butterflies, dragonflies, and other diurnal insects, more visual opsins improve color discrimination (Briscoe, 2008; Feuda, Marlétaz, Bentley, & Holland, 2016; Futahashi et al., 2015; Lunau, 2014; Sharkey et al., 2017). In contrast, species occupying nocturnal and subterranean (aphotic) habitats often lose one or more opsins, reducing their capacity for color vision (reviewed in Feuda et al., 2016, Tierney et al., 2017; but see Kelber, Balkenius, & Warrant, 2003; Meyer‐Rochow, 2007; Somanathan, Borges, Warrant, & Kelber, 2008; White, Xu, Münch, Bennett, & Grable, 2008).

In addition to reduced spectral sensitivity, nocturnal insects often sacrifice spatial and temporal resolution in order to optimize total visual sensitivity under low‐light conditions (but see Kelber et al., 2011). Moths and some nocturnal beetles have evolved superposition eyes with rhabdoms that collect light from multiple facets, sacrificing resolution to gain up to 1,000× more sensitivity than apposition eyes of similar dimension (Cronin et al., 2014; Horridge, 1969). Some crepuscular bees, ants, and dung beetles have more recently transitioned to a nocturnal niche, and possess apposition eyes with larger lenses and wider rhabdoms that sum photons over time and/or space to increase sensitivity (Baird, Fernandez, Wcislo, & Warrant, 2015; Warrant & Dacke, 2011). Among dung beetles, larger eye size, smoother facets, and absence of screening pigment correlate to nocturnal activity (McIntyre & Caveney, 1998). Nocturnal bees, wasps, and ants have dorsal ocelli—simple eyes used to detect ambient light, movement, and/or orientation—that are significantly larger than those of their diurnal relatives (Narendra & Ribi, 2017; Somanathan, Kelber, Borges, Wallén, & Warrant, 2009; Warrant, Kelber, Wallén, & Wcislo, 2006). The ocelli of nocturnal bees and cockroaches are sensitive, but poor at perceiving fast movements; they also lack UV‐sensitive opsins, perhaps as an adaptation to their UV‐poor light environments (Berry, Wcislo, & Warrant, 2011); similarly, the ocelli of nocturnal ants may have lost polarization sensitivity in response to the loss of polarized skylight signals (Narendra & Ribi, 2017).

## 3 EFFECTS OF ALAN ON NOCTURNAL INSECTS

When considering the environmental impact of ALAN, it is vital to distinguish between astronomical and ecological light pollution (Longcore & Rich, 2004). The former, often referred to as “skyglow,” is the result of upwelling illumination of the night sky. Skyglow may spread to cover an area far beyond its origin and is not blocked by local terrain (Gaston et al., 2015). In contrast, ecological light pollution refers to the infiltration of point sources of light into habitats on the ground, which can affect local species without necessarily influencing night sky brightness. Although shielded light technology may ameliorate skyglow, this light may still impact local biodiversity. Similarly, important is the position of insects relative to light sources; downwelling light may affect species on the ground more than those in the air, while upwelling light (path lights, tree lights, *etc*.) is more likely to affect insects in flight.

To provide a comprehensive framework for understanding the ecological impacts of artificial light on nocturnal insects, we have organized the relevant literature into five categories, described below.

## 4 TEMPORAL DISORIENTATION

ALAN may cause *temporal disorientation*, desynchronization of organisms from their typical biorhythms. The vast majority of terrestrial species have circadian, circamensual, and/or circannual patterns of activity (foraging, reproduction, migration, *etc.*) that are synchronized to daily, monthly, and yearly light cycles, respectively. Specialized photoperiodic photoreceptors use external light signals (*Zeitgebers* or “time‐givers”) to entrain the internal clock to the outside environment (Numata, Miyazaki, & Ikeno, 2015; Numata, Shiga, & Morita, 1997). In nocturnal insects, daily emergence time and duration of feeding and courtship activity are dictated by internal clocks entrained by ambient light or temperature (Saunders, 2009; Tataroglu & Emery, 2014), while monthly or yearly cycles of eclosion, mating, and oviposition can be entrained by moonlight or day length cues (for a review of lunar entrainment, see Kronfeld‐Schor et al., 2013). If ALAN is sufficiently intense and/or sustained in time, and of a specific spectral composition, it can desynchronize the internal clock (reviewed by Saunders, 2012). For example, the pink bollworm *Pectinophora gossypiella* can be artificially entrained by monochromatic yellow light emitted by low‐pressure sodium (LPS) lamps (Pittendrigh & Minis, 1971), and diurnal *Bombus terrestris* bumblebees by UV illumination (Chittka , Stelzer, & Stanewsky, 2013). Desynchronization reduces reproductive fitness of *Drosophila melanogaster* fruit flies (Xu, DiAngelo, Hughes, Hogenesch, & Sehgal, 2011, see also McLay, Nagarajan‐Radha, Green, & Jones, 2018) and has the potential to disrupt vital biological processes in other taxa (Dominoni, Borniger, & Nelson, 2016; Gaston et al., 2013; Saunders, 2012).

Exposure to artificial light in the laboratory inhibits a variety of biological processes in nocturnal insects (e.g., Botha, Jones, & Hopkins, 2017), possibly due to a buildup of melatonin (Honnen, Johnston, & Monaghan, 2016; Jones, Durrant, Michaelides, & Green, 2015). In moths, constant light can inhibit female sex pheromone release (Fatzinger, 1973; Geffen, Groot, et al., 2015; Sower, Shorey, & Gaston, 1970), reduce male attraction (Geffen, Eck, et al., 2015), induce male sterility (Bebas, Cymborowski, & Giebultowicz, 2001; Giebultowicz, 2001; Riemann, Johnson, & Thorson, 1981), and disrupt female oviposition (Yamaoka & Hirao, 1981). Because ALAN rarely completely conceals natural day–night cycles (Longcore & Rich, 2004; but see Honnen et al., 2016), it is unclear how these findings apply to natural populations. If skyglow is sufficiently constant and intense, some animals may respond by adopting around‐the‐clock activity patterns, as polar animals do during seasons with continuous light (Bloch, Barnes, Gerkema, & Helm, 2013). Even low‐level ALAN increases perceived day length (Kyba, Ruhtz, Fischer, & Hölker, 2011a), and could thereby induce seasonal polyphenism in stink bugs (Niva & Takeda, 2003) and aphids (Hardie, 2010; Sanders et al., 2015), or alter the calling songs of katydids (Whitesell & Walker, 1978). Low‐level ALAN is known to accelerate development in a range of insect taxa (Kehoe, Cruse, Sanders, Gaston, & Veen, 2018, van Geffen, Grunsven, Ruijven, Berendse, & Veenendaal, 2014, but see Durrant, Botha, Green, & Jones, 2018), with varying effects on fitness.

ALAN has been shown to conceal monthly and seasonal regimes of lunar sky brightness in urban areas (Davies, Bennie, Inger, Ibarra, & Gaston, 2013; Kyba, Ruhtz, Fischer, & Hölker, 2011b), and this could affect circamensual and circatidal rhythms of certain insect species (but see Satoh, Yoshioka, & Numata, 2008). Some populations of the mayfly *Povilla adusta* eclose 2 days after the full moon, which provides increased visibility in which to court, copulate, and oviposit (Corbet, Sellick, & Willoughby, 1974). Intertidal midges *Clunio marinus* (Neumann, 1989) and *Pontomyia oceana* (Soong, Lee, & Chang, 2011) use moonlight cues to synchronize activities (e.g., eclosion, mating, and oviposition) during intertidal periods. The corn earworm *Helicoverpa zea *will not mate unless its eyes are dark‐adapted to ambient light below 0.05 lux, similar to that produced by a quarter moon (Agee, 1969). Artificial light also suppresses *H. zea* oviposition, perhaps because this behavior is synchronized to the lunar cycle (Nemfc, 1971). Masking of lunar cycles by ALAN may disrupt these vital circamensual rhythms.

In addition to disrupting natural rhythms, ALAN can desynchronize ecological interactions of mutualist species. Different species have evolved to use either day length and temperature as *Zeitgebers* (Bale et al., 2002; Jayatilaka, Narendra, Reid, Cooper, & Zeil, 2011). Because ALAN alters day length but not temperature, it can lead to ecological mismatches. For example, the nightly foraging activity of the nocturnal carpenter bee *Xylocopa tranquebarica* is triggered by twilight and coincides with the opening of night‐blooming flowers (Somanathan et al., 2008). By delaying the onset of foraging in this bee (a “phase shift”; Pittendrigh, 1993), ALAN may disrupt this vital pollination mutualism, especially if flower opening is entrained by ambient temperature instead of light (van Doorn & van Meeteren, 2003, see also Seymoure, 2018).

ALAN may also prolong the foraging activity of diurnal and/or crepuscular competitors, which generally become more active with increasing illumination (Kempinger, Dittmann, Rieger, & Helfrich‐Forster, 2009; Kronfeld‐Schor et al., 2013). Initiation of foraging activity in moths (Dreisig, 1980; Eaton, Tignor, & Holtzman, 1983; Riley, Reynolds, & Farmery, 1983) and crickets (Campbell, 1976) is triggered when ambient light intensity declines to a species‐specific level. Foraging activity of nocturnal and crepuscular bees is both initiated and inhibited by specific light levels (Dyer, 1985; Kelber et al., 2006). In dung beetle communities, temporal partitioning helps reduce competition. Each species is physiologically adapted to a particular temporal niche: Nocturnal dung beetles have larger eyes and greater body size, which reduces radiant heat loss on cold nights (Caveney, Scholtz, & McIntyre, 1995). Crepuscular insects that phase shift into nocturnal niches may experience cold stress and could be slow to adapt (Urbanski et al., 2012). In this way, the disconnect of temperature and light environment may further disrupt ecological interactions by shifting species into temporal niches in which they experience greater competition and/or lowered fitness.

## 5 SPATIAL DISORIENTATION

ALAN may also result in *spatial disorientation*, disrupting an organism's ability to navigate in three‐dimensional space. In nocturnal landscapes, the lack of visual information makes navigation difficult. The most consistently visible landmarks are the moon and stars, followed by the dim pattern of polarized moonlight produced by atmospheric filtering (Dacke, Byrne, Baird, Scholtz, & Warrant, 2011). These orientation cues vary predictably throughout the night and seasonally, and nocturnal insects often use the moon or stars to calculate navigational bearings (e.g., black carpenter ants, Klotz & Reid, 1993; earwigs, Ugolini & Chiussi, 1996; heart‐and‐dart moths, Baker, 1987; and harvester termites, Leuthold, Bruinsma, & Huis, 1976).

Sand hoppers use the moon to orient along beaches and are known to orient to artificial fiber optic moons in the laboratory (Ugolini, Boddi, Mercatelli, & Castellini, 2005). Other animals can navigate by stars alone (reviewed by Foster, Smolka, Nilsson, & Dacke, 2018). On moonless nights, *Noctua pronuba* yellow underwing moths orient with respect to the north star (Sotthibandhu & Baker, 1979), and *Scarabaeus satyrus* dung beetles use the Milky Way as a cue to guide themselves away from dung piles in a maximally efficient straight line (Dacke, Baird, Byrne, Scholtz, & Warrant, 2013). On moonlit nights, *S. satyrus* and its relative *Scarabaeus zambesianus* (Dacke, Byrne, Scholtz, & Warrant, 2004; Dacke, Nilsson, Scholtz, Byrne, & Warrant, 2003) navigate using polarized moonlight; other nocturnal insects, including wasps (Warrant et al., 2006), bees (Greiner, Cronin, Ribi, Wcislo, & Warrant, 2007; Warrant & Dacke, 2011), and crickets (Herzmann & Labhart, 1989), may do so as well. The bull ant *Myrmecia pyriformis* uses polarized moonlight in addition to visual landmarks in the surrounding terrain to navigate to and from its nest during nightly foraging trips (Narendra, Reid, & Raderschall, 2013; Reid, Narendra, Hemmi, & Zeil, 2011).

ALAN has potential to interfere with all these forms of nocturnal navigation in two ways: Ecological light pollution introduces new sources of light into the nocturnal landscape, which could be confused for the moon or stars (see Attraction for one example), while atmospheric light pollution reduces the visibility of existing cues. Skyglow dramatically reduces star visibility in urban areas (Falchi et al., 2016), and artificial lighting has been shown to disrupt polarization signals as well (Kyba et al., 2011b).

Some nocturnal insects possess highly sensitive eyes capable of color discrimination under minimal illumination, including the hawkmoth *Deilephila elpenor* (Kelber et al., 2003) and the nocturnal bee *X. tranquebarica *(Somanathan et al., 2008). Both *X. tranquebarica *and the nocturnal sweat bee *M. genalis* use optic flow and visual landmarks such as trees and flowers to navigate to and from their nests (Baird, Kreiss, Wcislo, Warrant, & Dacke, 2011; Warrant et al., 2004). Several nocturnal ants (Hölldobler & Taylor, 1983; Kaul & Kopteva, 1982; Klotz & Reid, 1993), bees (Warrant et al., 2006), and the shield bug *Parastrachia japonensis* (Hironaka, Inadomi, Nomakuchi, Filippi, & Hariyama, 2008) have been shown to navigate by canopy patterns, and the site‐specific shape of leaves and branches silhouetted against the bright night sky overhead. Urban skyglow associated with ALAN could increase this contrast, while artificial illumination beneath the canopy is likely to erase it entirely.

Nocturnal insects such as wasps and bees use their enlarged ocelli for navigation (Berry et al., 2011; Goodman, 1965), and direct illumination may cause navigation problems for these and other species. Because these insects adjust their wing angle so that the lighter half of their visual field is always overhead, upward‐directed illumination could cause a maladaptive response. Indeed, light‐reflecting mulching films are used in open crop fields to suppress the arrival of alate aphids, thrips, and whiteflies (Shimoda & Honda, 2013). Disoriented insects might wander into unsuitable habitat, and those without the ability to “beeline” back to their nests at the end of each night may fall prey to overheating, desiccation, or predation the next day.

## 6 ATTRACTION

Through positive phototaxis, many flying insects are attracted to ALAN (Verheijen, 1960). Some exhibit characteristic spiraling flight patterns, while others approach the light directly. Some orbit the light source, frequently changing their angular velocity and direction to remain within its vicinity (Muirhead‐Thompson, 1991), while others perch on or under the light, apparently stunned. Physiological and behavioral explanations of this phenomenon abound (see Nowinszky, 2004), and their explanatory power varies with species. The light compass theory (Baker & Sadovy, 1978; Sotthibandhu & Baker, 1979) suggests insects that orient themselves by maintaining a constant angle to light rays, historically emitted only by the moon or stars, will spiral into artificial light sources. Other theories involve the illusion of open sky (Goldsmith, 1990) or dark “Mach bands” at light–dark borders (Hsiao, 1973), or disorientation due to “dazzling” (Robinson, 1952, Verheijen, 1960, Hamdorf & Höglund, 1981; see Desensitization below).

Historically, light traps have been used by scientists to survey community composition, monitor beneficial insects (Nabli, Bailey, & Necibi, 1999), and control insect pest populations (e.g., Goretti, Coletti, Veroli, Giulio, & Gaino, 2011; Pawson, Watt, & Brockerhoff, 2009; Wallner & Baranchikov, 1992). The most common insect orders attracted to and captured in light traps are Diptera, Coleoptera, and Lepidoptera (Mikkola, 1972; van Grunsven et al., 2014; Wakefield et al., 2016). Light‐trapping equipment can differ from ALAN in important ways: Experimental light traps usually emit more short wavelengths, are often without glass shields (which filter UV), and are placed near the ground (Degen et al., 2016). However, experiments that vary the intensity and spectral composition of light traps can still offer insight into the potential effects of ALAN on positively phototactic insects. The impact of ALAN on negatively phototactic insects such as cockroaches and earwigs has not yet been well‐explored (Bruce‐White & Shardlow, 2011, but see Farnworth, Innes, Kelly, Littler, & Waas, 2018), despite a clear potential for adverse effects (see Siderhurst, James, & Bjostad, 2006).

Among the common positively phototactic insects, moths (Frank, 1988, 2006 ; MacGregor, Pocock, Fox, & Evans, 2015) and aquatic insects (Perkin, Hölker, & Tockner, 2014; Yoon, Kim, Kim, Jo, & Bae, 2010) are best studied. Comparative surveys have shown that, relative to their calculated visibility, short wavelengths are disproportionately attractive to many insects (Barghini & de Medeiros, 2012; Mikkola, 1972; see also Wakefield et al., 2016 for a discussion of infrared wavelengths). Although most insects can perceive short wavelengths (Briscoe & Chittka, 2001; Kelber & Roth, 2006), certain families of moths are more attracted to them than others (van Langevelde, Ettema, Donners, WallisDeVries, & Groenendijk, 2011; Somers‐Yeates et al., 2013, see also Wölfling, Becker, Uhl, Traub, & Fiedler, 2016). LPS lamps rarely attract moths (Plummer et al., 2016; Robinson, 1952; Rydell, 1992), even though most species can detect the yellow wavelengths they emit (Briscoe & Chittka, 2001; Mikkola, 1972). Some nocturnal insects are disproportionately attracted to polarized light sources as well (Danthanarayana & Dashper, 1986; see Recognition below).

About 30%–40% of insects that approach street lamps die soon thereafter (Eisenbeis, 2006), as a result of collision, overheating, dehydration, or predation (Minnaar, Boyles, Minnaar, Sole, & McKechnie, 2015; Yoon et al., 2010). The presence of foraging bats does not repel moths from ALAN sources (Acharya & Fenton, 1999), and under mercury vapor light, *Operophtera brumata* and *O. fagata* moths lacked their normal evasive responses to simulated ultrasonic bat signals (Svensson & Rydell, 1998). Depending on its placement, ALAN may also impede the movement of insects among habitat patches, lure them into bodies of water, or divert them into traffic (Frank, 2006). Insects not killed immediately may become trapped in a “light sink,” unable to forage (Langevelde, Grunsven, et al., 2017), search for mates, or reproduce—especially when different sexes are disproportionately attracted to ALAN, as is the case for many moth species (Altermatt, Baumeyer, & Ebert, 2009; Altermatt & Ebert, 2016; Degen et al., 2016; Frank, 1988; Garris & Snyder, 2010; see also Farnworth et al., 2018). Ecological traps that result in mortality or reproductive failure are predicted to lead to rapid population decline and ultimately extinction (Kokko & Sutherland, 2001; Robertson, Rehage, & Sih, 2013). Long‐term records confirm that positively phototactic macro‐moths (Langevelde, Braamburg‐Annegarn, et al., 2017) in lit habitats (Wilson et al., 2018) have undergone disproportionate declines in abundance over the past 50 years.

Perhaps due to selection, when newly eclosed moths from urban populations are tested under standardized conditions, they are less attracted to ALAN (Altermatt & Ebert, 2016). ALAN in urban settings may also be generally less attractive due to a reduction in background contrast (Frank, 2006), although one study comparing declines in macro‐moth abundance at light‐trap sites with and without artificial night sky brightness did not support this suggestion (Conrad, Warren, Fox, Parsons, & Woiwod, 2006, see also White, 2018).

## 7 DESENSITIZATION

The highly sensitive visual systems of nocturnal insects may not always function well in illuminated environments. Crepuscular and nocturnal *Myrmecia* ants are capable of flexible, rapid light adaptation (Narendra, Greiner, Ribi, & Zeil, 2016), and some even forage more effectively under illumination (Narendra et al., 2013). However, the photoreceptors of other insects, such as nocturnal flies, bees, and cockroaches, are saturated at modest light levels (see Honkanen et al., 2017). When exposed to too many photons at once, some insects may be temporarily dazzled or even permanently blinded (Stark, Walker, & Eidel, 1985). In *Gryllus bimaculatus* crickets, the photoreceptors of nocturnal adults show structural degeneration after exposure to bright UV light, while those of diurnal nymphs are less affected (Meyer‐Rochow, Kashiwagi, & Eguchi, 2002). If not bright enough to cause permanent damage, a discrete ALAN source may still temporarily blind a nocturnal insect to other orientation cues by inducing a rapid process of light adaptation (see Laughlin & Hardie, 1978), perhaps causing it to fly directly into said light (McGeachie, 1988; Robinson, 1952; Verheijen, 1960). In the hawkmoth *D. elpenor*, an 8‐s exposure to bright blue light can reduce visual sensitivity by two to three orders of magnitude in minutes (Hamdorf & Höglund, 1981); even 0.125 s of white light exposure can effectively blind cockroach ocelli for 15–20 s (Ruck, 1958). Should an affected insect escape into darkness, it may take hours for it to completely recover its original visual sensitivity (Bernhard & Ottoson, 1960). Light adaptation can also have behavioral consequences: When moths encounter yellow or green light above a certain brightness, light adaptation of their eyes suppresses nocturnal behaviors, including flying, foraging, and mating, a response that has been successfully used in pest control (Shimoda & Honda, 2013).

## 8 RECOGNITION

The effects of environmental illumination on the ability of nocturnal insects to recognize objects in their environment (conspecifics, predators, food plants, *etc.*) will depend on both the wavelength and intensity of the ALAN source under consideration. Nocturnal insects may be able to detect nearby objects more easily if the source emits (at sufficient intensity) wavelengths to which their visual systems are sensitive. However, some taxa may gain or lose their ability to discriminate colors, depending on the range of wavelengths emitted (Davies, Bennie, Inger, Ibarra, et al., 2013, but see Johnsen et al., 2006). As a result, ALAN has the potential to impede visual signaling and/or undermine camouflage (reviewed by Delhey & Peters, 2017). The body color of dusk‐active beetles is most apparent in a purplish light environment (Endler, 1993; Théry et al., 2008) and may become less visible to conspecifics under broad‐spectrum ALAN illumination. Similarly, the aposematic coloration of *Heliconius *butterfly wings, which is especially apparent in their typical light environments, may be obscured by ALAN (Seymoure, 2016). The UV emissions of mercury vapor lamps accentuate UV‐reflective markings on flowers and wings, which may benefit bees, moths, and other nocturnal insects sensitive to these signals (Kevan, Chittka, & Dyer, 2001). In contrast, illumination by LPS lamps could obscure these markers (Frank, 2006). Many aquatic insects use polarized light to locate suitable oviposition sites (reviewed by Horváth, Kriska, Malik, & Robertson, 2009, Perkin et al., 2014, Villalobos Jiménez, 2017). Artificial illumination of smooth dark surfaces such as asphalt simulates the polarization of light reflected off bodies of water, causing some aquatic insects to maladaptively oviposit on bridges or cars (Egri et al., 2017; Szaz et al., 2015), or to congregate on windows (Horváth et al., 2009; Kriska, Malik, Szivák, & Horváth, 2008).

Increased visibility is thought to benefit predators over prey (Kronfeld‐Schor et al., 2013; Youthed & Moran, 1969), but evidence of this phenomenon in arthropod systems is mixed (see Grenis, Tjossem, & Murphy, 2015; Skutelsky, 1996; Tigar & Osborne, 1999). By simulating bright moonlit nights every night of the month (Davies, Bennie, Inger, & Gaston, 2013), ALAN may reduce foraging time and increase the starvation risk of nocturnal prey insects (Schmitz, Beckerman, & O'Brien, 1997). Conversely, it may prolong the foraging activity of diurnal and crepuscular insects such as the sweat bee *Lasioglossum texanum* (Kerfoot, 1967), the desert ant *Veromessor pergandei* (Hunt, 1977), and the hemipteran *Nilaparvata lugens* (Riley, Reynolds, & Farrow, 1987), all of which engage in nocturnal foraging during the full moon.

## 9 COMMUNITY‐LEVEL IMPACTS

By altering the light environment experienced by nocturnal insects, ALAN can disrupt their temporal patterns, interfere with their spatial orientation, act as a fatal attraction, reduce their visual sensitivity, and alter foraging activity and species interactions. When populations of such abundant and ubiquitous organisms are disrupted (Gaston & Bennie, 2014; Kurvers & Hölker, 2015), entire communities will be affected (Davies et al., 2017; Davies, Bennie, Inger, Ibarra, et al., 2013; Sanders & Gaston, 2018). The population‐ and community‐level effects of ALAN have been relatively understudied, despite their great potential for use in predicting the future composition of artificially illuminated habitats; we summarize existing research below.

Insects that are attracted to ALAN sources are readily exploited by predators: Orb‐weaving spiders prefer artificially lit web sites (Enders, 1977), which may net them more prey (Heiling, 1999, but see Yuen & Bonebrake, 2017). Bats (Jung & Kalko, 2010; Minnaar et al., 2015; Rydell, 2006), birds (Robertson, Kriska, Horvath, & Horvath, 2010), and invasive cane toads (González‐Bernal, Greenlees, Brown, & Shine, 2016) congregate around streetlights and lit buildings for a similar reason. Usually, diurnal anole lizards and jumping spiders have been observed hunting for insects at night in artificially illuminated locations (Frank, 2009; Garber, 1978; Wolff, 1982). Moths frozen under illumination may provide stable search images that enable birds to recognize the camouflaged wing patterns of these species in other contexts (Frank, 2006). Decreases in moth abundance have a negative impact on nocturnal pollen transport (Fox, 2013; Frank, 1988; Knop et al., 2017; Macgregor et al., 2015; Macgregor, Evans, Fox, & Pocock, 2017), with cascading effects on populations of plants and insect herbivores.

Artificial illumination in urban areas may reduce the population persistence of nocturnal species by preventing movement between habitat patches (Farnworth et al., 2018; Gaston & Bennie, 2014; Guetté et al., 2018). For example, moths attempting to cross road networks were impeded by a line of closely spaced street lights (Degen et al., 2016). In a riparian ecosystem, emerging aquatic insects were drawn to artificially illuminated patches rather than the surrounding habitat, potentially reducing nutrient exchange and species dispersal (Manfrin et al., 2017). At the same time, illumination of the water may increase predation risk for invertivorous fish, reducing predation on aquatic insects and leading to locally increased insect abundance (Manfrin et al., 2017). Riparian predators respond to increased prey availability by congregating around light sources (Meyer & Sullivan, 2013; Perkin et al., 2011).

Several studies have noted an influx of predatory and scavenging arthropods into lit areas (Davies, Bennie, & Gaston, 2012; Šustek, 1999), though this response appears to be taxon‐specific (Eccard, Scheffler, Franke, & Hoffmann, 2018; Manfrin et al., 2017; Meyer & Sullivan, 2013; van Grunsven, Jähnichen, Grubisic, & Hölker, 2018). Broad‐spectrum LED lights in combination with urban heat reduced pea aphid populations by increasing visibility and lengthening the activity period of their visually oriented coccinellid predators (Miller et al., 2017); however, in similar experiments, bright illumination decreased or did not affect rates of parasitism by parasitoid wasps (Kehoe et al., 2018; Sanders et al., 2015, see Sanders, Kehoe, Cruse, Veen, & Gaston, 2018). The introduction of artificial light and noise causes parasitic frog‐biting midges to be unable to locate and feed from their túngara frog hosts (McMahon, Rohr, & Bernal, 2017). Herbivorous insects may additionally be affected by changes in their food plants (reviewed by Vänninen, Pinto, Nissinen, Johansen, & Shipp, 2010). For example, long‐wavelength ALAN reduced the population sizes of pea aphids by inhibiting flowering in their host plant via the phytochrome pathway (Bennie, Davies, Cruse, Inger, & Gaston, 2015), and light from high‐pressure sodium (HPS) lamps increased plant toughness and decreased the mass of cutworm larvae (Grenis & Murphy, 2018). The resultant absence of midges, pea aphids, and other prey insects in urban areas is likely to have widespread effects on populations of their host plants, pollinated plants, and predators.

## 10 EFFECTS OF ALAN ON BIOLUMINESCENT INSECTS

Firefly beetles (Coleoptera: Lampyridae) comprise the most widespread and diverse group of bioluminescent species on land. The approximately 2000 lampyrid species, all of which glow aposematically as larvae (Branham & Wenzel, 2003), enjoy a worldwide distribution. In many adult fireflies, either one or both sexes employ bioluminescence as a courtship signal (Lloyd, 2008). Other bioluminescent insect taxa, primarily click beetles (Coleoptera: Elateridae), railroad worms (Coleoptera: Phengodidae), and fungus gnats (Diptera: Keroplatidae), employ bioluminescence as an aposematic signal or predatory lure (Meyer‐Rochow, 2007, Redford, 1982). All of these essential signals may be masked by ALAN, which has been suggested as one of several factors contributing to a worldwide decline in firefly populations (Khoo, 2014; Lewis, 2016; Lloyd, 2006). Recent studies on bioluminescent ostracods (Gerrish, Morin, Rivers, & Patrawala, 2009) and fungus gnats (Merritt & Clarke, 2013; Mills, Popple, Veidt, & Merritt, 2016) have shown inhibitory effects of artificial light on signaling activity, raising similar concerns. In this section, we briefly discuss firefly visual ecology then use our framework to examine how ALAN may affect the courtship and reproductive success of these and other bioluminescent insects.

## 11 FIREFLY VISION AND BIOLUMINESCENCE

Bioluminescent fireflies employ a diverse range of courtship signaling systems as part of their sexual communication (Lewis, 2009; Takatsu, Minami, Tainaka, & Yoshimura, 2012). In some taxa, sedentary females produce long‐lasting glows that attract flying males, the latter sometimes incapable of producing light. In other taxa, including most North American species, both sexes use flash signals—discrete bursts of light—to communicate with potential mates. North American *Photinus* fireflies engage in courtship dialogs that involve precisely timed flash signals encoding species identity and sex. Typically, sedentary females respond to advertisement flashes emitted by flying males. In congregating South‐East Asian species, however, clusters of stationary males emit synchronous flashes to attract flying females.

Bioluminescence can be a highly efficient visual signal: against a black background, its contrast is effectively infinite, and the distances across which it can be perceived limited only by habitat structure and the visual sensitivity of the receiver (Cronin et al., 2014). However, fireflies do not always signal against a black background. While nocturnal species initiate courtship flashing long after nightfall, crepuscular species become active shortly after sunset (Lloyd, 1966). Some species flash in shady patches during the daytime (Vencl, Luan, Fu, & Maroja, 2017). The color of firefly bioluminescence varies as follows: Among crepuscular species, flashes are generally yellower compared to the greener flashes emitted by nocturnal fireflies (Seliger, Lall, Lloyd, & Biggley, 1982); this is thought to maximize signal contrast against the green foliage dominating the background at dusk. However, signal color also shows intraspecific variation, perhaps related to differences in habitat type (Hall, Sander, Pallansch, & Stanger‐Hall, 2016).

Due to their temporally restricted courtship activity periods, fireflies are highly sensitive to ambient light cues indicating time of day. When ambient light intensity descends to a certain species‐specific threshold, courtship signaling begins (Buck, 1937a; Table 1). Intracerebral ocelli described from Japanese *Luciola cruciata *and *Luciola lateralis* fireflies may assist in entraining this circadian behavior (Hariyama, 2000). *Lampyris noctiluca* females usually begin emitting courtship signals when ambient light levels fall below 1.3 lux and are never active above 15 lux; for *Photuris congener* males, these values are 0.25 and 2.5 lux, respectively, reflecting the shorter duration of twilight in their habitat (Dreisig, 1975). Ambient light intensity also affects other aspects of courtship behavior. In crepuscular *Photinus* species, males fly higher as light levels decline, and when passing under the shade of trees (Lewis & Wang, 1991; Lloyd, 2000).

**Table 1:** Effect of ambient light on flash activity of various firefly species^a^

Species	Flash (lux)	No flash (lux)	Location	References
*Aspisoma lineatum*	0.85		Southeast Region, Brazil	Hagen and Viviani (2009)
		>0.05		Hagen et al. (2015)
*Apisoma physonotum*	<0.2			Hagen and Viviani (2009)
		>0.05		Hagen et al. (2015)
*Apisoma sp2*	<0.2			Hagen and Viviani (2009)
*Apisoma sp4*	<0.2			Hagen and Viviani (2009)
*Bicellonychia lividpennis*	4.5			Hagen and Viviani (2009)
*Bicellonychia ornaticollis*	<0.2			Hagen and Viviani (2009)
*Cratomorphus concolor*	<0.2			Hagen and Viviani (2009)
*Cratomorphus sp4*	<0.2			Hagen and Viviani (2009)
*Lampyris noctiluca *(♀)	1.3	10	Ebeltoft, Denmark	Dreisig (1975)
	0.28			Dreisig (1975)
*Lampyris noctiluca* (larva)	6.85			Dreisig (1975)
*Luciola italica*	0.25	0.47 ± 0.26	Turin, Italy	Picchi, Avolio, Azzani, Brombin and Camerini (2013)
*Photinus interdius *(diurnal)	339.82 ± 88	>1,000	Darien Province, Panama	Vencl, Luan, Fu, and Maroja (2017)
*Photinus pyralis*		210–320	Laboratory	Buck (1937a)
	301.24 ± 89.07		Clarke County, Virginia, USA	Firebaugh and Haynes (2016)
*Photinus sp1*	<0.2	>0.234	Southeast Region, Brazil	Hagen et al. (2015)
		>1.5		Hagen and Viviani (2009)
*Photinus *spp.	1.2		Piedmont Region, Maryland, USA	Costin and Boulton (2016)
*Photinus umbratus*	1		Highlands County, Florida, USA	Dreisig (1975)
	4			Dreisig (1975)
*Photuris "A"*	0.38			Dreisig (1975)
*Photuris congener*	0.25	2.5		Dreisig (1975)
*Photuris pennsylvanica*		160,000	Laboratory	Harvey (1926)
*Photuris missouriensis*		3,800	Laboratory	Case and Trinkle (1968)
*Photuris versicolor *(♀)			Highlands County, Florida, USA	Dreisig (1975)
		301.24 ± 89.07	Clarke County, Virginia, USA	Firebaugh and Haynes (2016)
*Pteroptyx maipo*	0.2–0.3		Tin Shui Wai, Hong Kong	Yiu (2012)
*Pteroptyx valida*	7–14		Samut Prakan Province, Thailand	Prasertkul (2018)
*Pyrogaster moestus*		>0.05	Southeast Region, Brazil	Hagen et al. (2015)
*Pyrogaster sp1*	<0.2			Hagen and Viviani (2009)

a “Flash” column gives ambient light levels shown to be dim enough to induce bioluminescence for each species; “No flash” gives ambient light levels shown to inhibit firefly flash activity.

The superposition compound eyes of fireflies are finely attuned to conspecific signals: They are highly sensitive, but only to a narrow range of wavelengths. To date, only UV‐ and long‐wavelength‐sensitive (UVS and LWS) opsin genes have been isolated from fireflies (Martin, Lord, Branham, & Bybee, 2015; Sander & Hall, 2015), although blue sensitivity has been observed in both North American and Japanese species using electroretinography (ERG) recordings (Eguchi, Nemoto, Meyer‐Rochow, & Ohba, 1984; Lall, Lord, & Ovid Trouth, 1982; Lall, Strother, Cronin, & Seliger, 1988). The peak sensitivity of the LWS photoreceptor generally corresponds to the peak wavelength of signals produced by that species (Sander & Hall, 2015). Signal reception is further tuned using filter pigments: In species that emit yellow bioluminescence, reddish filter pigments narrow visual sensitivity to the yellow region of the spectrum by blocking out green wavelengths (Booth, Stewart, & Osorio, 2004; Cronin, Järvilehto, Weckström, & Lall, 2000). This screening could allow some lampyrids to communicate visually even within light‐polluted habitats. However, current evidence suggests that ALAN does have a demonstrable impact on firefly signaling (Table 2).

**Table 2:** Effect size of experimental studies examining impact of ALAN on firefly courtship

Species	Treatment (ALAN type)	Metric	Intensity (lux)	Effect size (G's Δ)	Study
*Lampyris noctiluca*	High‐pressure sodium street lamps	Green LED lure (# of trapped males)	L1: 46–64	L1: −0.74	Ineichen and Rüttimann (2012)
			L2: 0.1–0.4	L2: −0.97	
*Lampyris noctiluca*	Incandescent flashlight	Green LED lure (# of trapped males)	L1: 0.3	L1: −0.37	Bird and Parker (2014)
			L2: 0.18	L2: −0.37	
			L3: 0.09	L3: −0.23	
			L4: 0.07	L4: −0.02	
*Photinus sp1*	Multi‐metal vapor floodlights	Transect count (# of flashing individuals)	T1: 4.45	T1: −0.25	Hagen et al. (2015)
			T2: 1.5	T2: −0.22	
			T3: 0.05	T3: −0.17	
*Photuris versicolor*	White LED floodlights	Flash count (flashes/min)	301 at plot center	−0.459	Firebaugh and Haynes (2016)
*Photinus pyralis*		Flash count (flashes/min)	301 at plot center	NS	
		Flash count, tethered females (15 min total)	167.21 on average	−0.617	
*Photinus *spp.	Mercury vapor bulb	Flash count (flashes/min)	1.2 at plot edge	−0.653	Costin and Boulton (2016)

Note

Because all studies involve comparison of groups of the same size (e.g., firefly populations before and after ALAN exposure), Glass's Δ is an appropriate estimate of effect size, as it uses only the standard deviation of the control group (Kline, 2013). NS indicates that no significant effect on firefly courtship activity was observed.

Studies in urban and rural regions in São Paulo, Brazil, have found that several lampyrid species are limited to areas with ambient light levels below 0.2 lux, approximately equivalent to that of the night sky during a full moon (Hagen & Viviani, 2009; Viviani, Rocha, & Hagen, 2010). However, the crepuscular species *Aspisoma lineatum *and *Bicellonychia lividipennis* were found signaling near sodium vapor lamps, in areas with illumination measuring 0.85 and 4.5 lux, respectively; note that *B. lividipennis* emerges early in the evening when ambient light levels (without ALAN) reach about 4.5 lux. Similarly, surveys of *Luciola italica* in Turin, Italy, have found populations concentrated in dimly illuminated areas, with a negative correlation between firefly abundance and certain indices of urbanization (Picchi et al., 2013). In descriptive studies such as these, the effects of ALAN are confounded by many other variables associated with urbanization. It is also unclear whether population loss occurs due to movement away from illuminated habitats or to reduced reproductive success within urban populations.

Below, we summarize all available evidence concerning the impact of ALAN on fireflies, organized according to the five categories of impact described earlier. Many of these findings may be applicable to all bioluminescent insects, although data on other taxa are sparse. Should these insects be unable to cope with the following challenges, their survivorship, foraging success, and mating success—and therefore population persistence—will be reduced.

### 11.1 Temporal disorientation

ALAN has the potential to disrupt both larval and adult activity cycles, but little is known about the magnitude of this impact. *Pyractomena borealis* larvae kept indoors under continuous light pupated several months earlier than anticipated (Lloyd, 2006), but whether this occurs in natural populations remains unclear. Although adult fireflies rarely live long enough to express circamensual rhythms of activity, *L. noctiluca *larvae in the field have been shown to hide during the full moon (Gunn & Gunn, 2012), and may reduce their foraging activity if night sky brightness is increased by skyglow.

Nightly onset of courtship activity by adult fireflies is determined by ambient light intensity. *P. pyralis* adults begin flashing earlier in the evening on cloudy days (Buck, 1937a), although the opposite is predicted for urban areas, where reflection of ALAN makes the sky brighter on cloudy days than on clear ones (Kyba et al., 2011a). In light‐polluted habitats, the flight periods of crepuscular species might be extended into those normally occupied by nocturnal species. If males or females are responsive to heterospecific signals, this could impact their reproductive success by reducing dialog efficiency and/or increasing the frequency of heterospecific matings. Nocturnal *Photuris* fireflies are a major predator of other firefly species (Eisner, Goetz, Hill, Smedley, & Meinwald, 1997; Eisner, Wiemer, Haynes, & Meinwald, 1978; Lloyd, 1997). If ALAN causes crepuscular species to extend their courtship into the nocturnal niche occupied by *Photuris*, they may experience higher predation rates. It has been posited that, over evolutionary time, predation by nocturnal *Photuris* fireflies drove some fireflies into crepuscular and diurnal niches (Deyrup et al., 2017; Gronquist et al., 2006).

### 11.2 Spatial disorientation

ALAN may disorient fireflies that navigate with respect to the sun or moon, such as the larvae of *P. borealis*, which choose an aerial pupation site located on the southern side of trees to maximize their exposure to direct sunlight (Gentry, 2003). However, little is known about the balance of positive and negative phototaxis in fireflies. In *Photinus* fireflies, males produce courtship signals while searching within their habitat, and immediately orient toward a female when they detect her response flash. Because these fireflies do not appear capable of distinguishing between small signals that are nearby and large signals farther away (Cratsley & Lewis, 2003), they could possibly mistake artificial lights with certain emission spectra for receptive conspecifics (see below).

### 11.3 Attraction

In the European glowworm *L. noctiluca*, flying non‐bioluminescent males are attracted to larviform females that glow steadily from perches on raised display sites. Numerous experimental studies conducted with this species have employed glowing light lures to elucidate their signaling system (e.g., Bird & Parker, 2014; Mikkola, 1972; Schwalb, 1961). By measuring the attraction of males to different LEDs or chemiluminescent lures, these studies have described male preferences for the color and spatial pattern of female glow signals, and have demonstrated male attraction to light traps that emit yellow‐green light (reviewed by De Cock, 2009).

Although *L. noctiluca *is geographically widespread in Europe, its populations have been declining for some time (Gardiner, 2011). One potential contributing factor could be that males are more attracted to certain point sources of ALAN than they are to conspecific female glow signals. In North Wales, Bek (2015) reported significant attraction of male *L. noctiluca *to LPS street lamps, compared to HPS, LED, or unlit lamps: The vast majority of males (556 of 564) were found under LPS lamps, while none were found below LED lamps or those that had been switched off. A notable difference between LPS and LED bulbs is that the latter emit a large percentage of their light in blue wavelengths; binary choice experiments on *L. noctiluca* suggest that the addition of blue wavelengths significantly decreases male attraction to light lures (Booth et al., 2004).

Though *L. noctiluca *is not representative of all firefly species, other glowworms may also be attracted to artificial lights. In Uganda, Bowden and Church (1973) found multiple *Lamprigera* specimens caught in a Robinson trap illuminated by a mercury vapor bulb. In Rwanda, *Diaphanes* males were collected from a light trap emitting red light (Pacheco, Martin, & Bybee, 2016). *Pleotomus* and *Pleotomodes* glowworms are also attracted to light traps, including those exclusively emitting UV wavelengths (Faust, 2017; Lloyd, 2006).

Beyond glowworms, other firefly species could also be attracted to artificial light sources. Male *Pteroptyx* fireflies in South‐East Asia congregate in particular display trees and flash together *en masse*, and may be attracted to flashing string lights imitating these mating congregations (Cratsley, Prasertkul, & Thancharoen, 2012; Thancharoen, Srinual, & Laksanawimol, 2017). If these light sources draw individuals away from traditional display sites or differentially attract males and females, they could disrupt *Pteroptyx* courtship and reduce mating success.

### 11.4 Desensitization

Due to their highly sensitive visual systems (Horridge, 1969), fireflies may be vulnerable to blinding by bright ALAN sources. Numerous previous studies have used ERG recordings to measure firefly spectral sensitivity, by observing the level of electrical activity in the eye triggered by exposure to small point sources of light (e.g., Lall, 1981; Lall et al., 1982, 1988; Lall, Chapman, et al., 1980; Lall & Lloyd, 1989). These studies show that the compound eyes of *Photinus* fireflies can take several hours to become fully dark‐adapted (Lall, 1993); intermittent light exposures may decrease resulting gains in visual sensitivity, but the duration and intensity of exposure sufficient to cause a complete reversal to the light‐adapted state, let alone to cause dazzling and/or blinding, have yet to be investigated (A. Lall, pers. comm.). Nonetheless, it appears likely that bright sources of ALAN such as LED street lamps could at minimum slow the dark adaptation process. This may disproportionately diminish long‐wavelength sensitivity in female fireflies (Oba & Kainuma, 2009), reducing their ability to recognize potential mates.

### 11.5 Recognition

While some background illumination is often present in their signaling milieu, especially for crepuscular firefly species, artificial illumination could reduce courtship success by interfering with the perception of male signals by receptive females, or vice‐versa. In addition, females might be less responsive to male signals that they perceive as dimmer under ALAN. Lloyd (2006) suggested that monochromatic yellow light from LPS lamps might disproportionately impact the signal exchange of yellow‐flashing (crepuscular) species. Because broad‐spectrum illumination from white LED street lamps includes many wavelengths, these ALAN sources may obscure the bioluminescent signals of green‐flashing (nocturnal) firefly species as well.

Several recent field studies have examined how ALAN affects firefly flash activity and courtship behavior (Table 2). Ineichen and Rüttimann (2012) assessed the impact of HPS street lighting on *L. noctiluca* males in suburban Switzerland. They compared male attraction to LED lures simulating the glows of conspecific females, placed either under (46–64 lux) or between (0.1–0.4 lux) HPS street lamps. Significantly, more males were attracted to lures in darker locations between the HPS street lamps. Perhaps males were unable to detect the lures in the highly illuminated surroundings, or perhaps males prefer signals with greater contrast against the background (Hopkins, Baudry, Candolin, & Kaitala, 2015). In either case, these results suggest HPS street lighting interferes with *L. noctiluca* courtship communication. Ineichen and Rüttimann (2012) noted that female display sites appeared to be uniformly distributed with respect to street lighting. *L. noctiluca* females select their display sites during daytime as larvae and rarely relocate in their flightless adult form (Tyler, 2013). Thus, females located underneath ALAN sources are likely to experience lower mating success.

In another observational study on a university campus in Sorocaba, Brazil, Hagen, Santos, Schlindwein, and Viviani (2015) found that courtship flashing by several firefly species was affected by multi‐metal vapor spotlights illuminating an outdoor sport court. On nights when the spotlights were turned on, flash activity by the most common species, *Photinus* sp1, was significantly reduced. ALAN reduced the number of flashing individuals encountered along transects that were both directly and indirectly illuminated by the spotlights; *Photinus* sp1 flashed only below measured light intensities of 0.23 lux. While this and other studies clearly demonstrate that ALAN reduces flash activity (Table 1), its impact on firefly mating success remains unknown.

Experimental field studies of ALAN are useful for removing confounding variables associated with urbanization. Working in an undisturbed British chalk grassland, Bird and Parker (2014) introduced a point source of light and then measured attraction of *L. noctiluca *males to glowing LED lures. Lures were placed 0.5, 1.0, 1.5, and 2 m from an upward‐directed filament bulb flashlight (its emission spectra resembling that of a mercury vapor lamp) with measured intensities of 0.3–0.07 lux. Compared to a non‐illuminated control, levels of ALAN as low as 0.09 lux significantly reduced the number of males attracted to simulated female glows. When ambient illumination was brighter than 0.18 lux, none of the males approached the simulated females.

Although both studies showed that ALAN can interfere with the ability of male *L. noctiluca* to locate females, Bird and Parker (2014) found effects at much lower ALAN levels compared to Ineichen and Rüttimann (2012). Several explanations are possible. Bird and Parker attributed this difference to the orientation of the ALAN source, suggesting that upward‐directed light is more likely to dazzle males or reduce trap visibility than downward‐directed street lamps. The studies also differed in ALAN type as well as design of the female lures. Furthermore, urban and suburban firefly populations may have adapted to cope with higher levels of ALAN. Male choice is another potential factor: In both studies, males chose the comparatively brightest lures of those presented.

Firebaugh and Haynes (2016) conducted an experimental study of the impact of ALAN on the flash activity of both male and female fireflies at a rural site in Virginia, USA. They established four pairs of 20‐m‐diameter plots, placing a downward‐facing LED floodlight (301 lux at plot center) at the center of each plot. The flash activity of *Photuris versicolor*, a nocturnal species, declined significantly in the illuminated plots compared to non‐illuminated control plots. However, no difference in flash activity was detected for males of *P. pyralis*, a crepuscular species. In addition, ALAN did not alter the timing of the nightly onset of *P. pyralis* flash activity.

In a separate experiment, the authors tethered *P. pyralis *females to display platforms and observed the impact of LED floodlights (approximately 167 lux) on male approach frequency and flash count, as well as on the frequency of female response flashes. Although ALAN again had no significant effect on the flash activity of *P. pyralis *males, it significantly reduced the female response rate. This differential effect on male and female flash behavior could be due to the downward directionality of the ALAN source. Flying males may be able to detect a female flashing below them, even against more brightly illuminated vegetation. However, females perch close to the ground and look upward to detect males, typically against a dark sky. Therefore, the females may have been unable to detect male signals viewed against the downward‐facing ALAN. Alternatively, since *P. pyralis* females are known to prefer brighter simulated flashes (Vencl & Carlson, 1998), they may be less responsive to male signals that, when set against an illuminated background, appear less bright.

In an experimental field study in rural Maryland, USA, Costin and Boulton (2016) introduced ALAN using a mercury vapor bulb (1.2 lux at plot edge). At each of the six sites starting 30 min after sunset, they counted firefly flashes for 30 min one night, then added ALAN, and repeated the count the next night. On nights when ALAN was introduced, the authors found a significant decline in flash rates. Multiple species were present at each site, with the crepuscular *Photinus marginellus* most frequently observed. In contrast, Firebaugh and Haynes (2016) found no effect of ALAN on flash activity of *P. pyralis*. The discrepancy between these studies could reflect differing emission spectra of their artificial light sources, and/or differences between firefly species. Although both are crepuscular, *P. marginellus *emerges slightly later in the evening (Lloyd, 1966) and might be more sensitive to ALAN than *P. pyralis*. Because control counts were always made on the night preceding ALAN treatment at each site, the results may also reflect temporal changes in firefly abundance.

Yiu (2012) performed a similar experiment on synchronously flashing males of a Hong Kong firefly *Pteroptyx maipo*, introducing a compact fluorescent lamp for several minute intervals and counting flashes before and during exposure. ALAN illumination (0.21–2.0 lux) significantly decreased the average flash frequency of this species, and these decreases were reversed when the lamp was switched off. However, during a survey of *Pteroptyx malaccae* and *Pteroptyx valida* in Thailand, Prasertkul (2018) found robust congregations of both species flashing within close proximity to white fluorescent street lamps (up to 7–14 lux), suggesting that ALAN does not prevent aggregation or courtship flashing in these species.

In contrast to the complex light environments and shifting conditions typical of field experiments, laboratory experiments allow for more precise manipulation of ambient light levels. Early light exposure experiments conducted in the laboratory demonstrated that sufficiently bright light (from 50 to 1,000 lux) completely inhibits bioluminescent signaling in several firefly species (Table 1). More recently, Thancharoen (2007) exposed pairs of the Thai firefly *Sclerotia (formerly Luciola) aquatilis* to different intensities of fluorescent lighting (0.05, 0.1, 0.2, and 0.3 lux) and observed their mating behavior. Artificial illumination prolonged courtship, mounting, and mating duration in this species. Though all pairs ultimately mated successfully in the laboratory, ALAN could reduce mating success under field conditions by increasing the difficulty of locating a mate.

Using monochromatic LEDs, Owens, Meyer‐Rochow and Yang (2018) investigated the impact of ambient light on the alarm flash behavior of an aquatic Taiwanese firefly, *Aquatica ficta*. Short‐wavelength light (444–533 nm) caused males to flash more brightly, but less frequently, while long‐wavelength light (597–663 nm) had no significant effect on alarm flash behavior. These results indicate that fireflies can respond to increased background illumination by producing light signals of greater intensity. These results also suggest that, at least for this species, long‐wavelength artificial light (>597 nm, amber to red) is less disruptive than short‐wavelength (444–533 nm, violet to green) ALAN (but see Buck, 1937b, Pacheco et al., 2016).

## 12 CONCLUSIONS

Widespread nocturnal artificial illumination radically disrupts the habitats of night‐active species. Nocturnal and crepuscular insects are abundant and important components of these ecosystems. Thus, the impact of ALAN on insect fitness and abundance can provide a useful metric of overall ecosystem disturbance. The potential effects of ALAN on insects can be categorized as temporal disorientation, spatial disorientation, attraction, desensitization, and recognition. The severity of impact will depend on the degree of overlap between the spectral sensitivity of the insect in question and spectral emission (and intensity) of the particular ALAN source (Gaston et al., 2015). Recently, many urban areas have begun to phase out monochromatic long‐wavelength LPS lamps in favor of broad‐spectrum white LED lighting. This spectral shift represents an ecological experiment on a global scale, with potentially devastating results.

One recommendation is of paramount importance: future studies concerning the impact of ALAN on nocturnal organisms should employ more objective, less anthropocentric methods for measuring light. Light meter measurements in lux are adjusted for human luminous sensitivity and can be greatly affected by differences in distance from and angle to a light source. Insects often occupy discrete microhabitats within larger light environments. Therefore, researchers should strive to objectively measure how light varies within, as well as among, nocturnal habitats.

Among night‐active insects, bioluminescent fireflies are conspicuous and charismatic flagship species that can attract support for conservation efforts aimed at minimizing excess nocturnal illumination in urban areas. As a result, recent studies conducted in several geographic locations have focused on the impact of ALAN on fireflies. Laboratory and field studies reviewed here demonstrate that ambient light can inhibit the courtship flashing of several firefly species. ALAN reduced courtship signaling by both sexes in a North American firefly and reduced the ability of male European glowworms to locate females. Laboratory studies testing the effects of monochromatic light found that an Asian firefly is capable of increasing its flash intensity in response to ALAN and that long wavelengths (amber to red) were least disruptive.

This review also identifies several key gaps in our knowledge concerning the impacts of ALAN on fireflies, and highlights several important directions for future research. We currently know little about temporal disorientation or desensitization by ALAN, and these effects deserve further study. For example, comparative studies of firefly phenology and nightly emergence times could reveal the degree to which ALAN delays and/or shortens the temporal scope of courtship activity. Furthermore, despite evidence from several firefly species that ALAN interferes with mate location, it remains to be seen whether this disruption has consequences for mating and reproductive success. We also need information on how these behavioral impacts translate into longer‐term effects on population size and persistence.

Fireflies may be able to successfully cope with ALAN in various ways, including increased dispersal or evolutionary adaptation. Can fireflies move away from artificial lights toward darker habitat? We currently know little about dispersal abilities in either the adult or larval stages for any firefly species. While evidence suggests that the colors of firefly bioluminescence are under selection to maximize signal contrast in different natural light environments (Hall et al., 2016; Lall, Seliger, et al., 1980), whether ALAN selects for genetic changes in firefly signals remains unknown. Comparison of urban and rural populations may provide insight into adaptations that allow fireflies to cope with high levels of ALAN.

It will also be important to investigate how different intensities and colors of ALAN affect the courtship signaling of different firefly species. While physiological studies (e.g., ERGs) have revealed variation among species in their spectral sensitivity, the impact of different wavelengths on courtship success has yet to be determined. One particularly important aspect of this issue is understanding how the blue wavelengths emitted by commercial LED street lamps influence courtship signals and mate location across different firefly species. This research can help guide development of new lighting technology that balances the need for public safety, energy efficiency, and conservation, and inform policy recommendations for firefly‐friendly ALAN sources that can be deployed on public, commercial, and private lands in or near firefly habitat.

## CONFLICT OF INTEREST

None declared.

## AUTHORS CONTRIBUTION

ACSO conceived and drafted the first version of this manuscript and performed all data analyses. Both authors contributed significantly to revisions and have given final approval of the version to be published.

## DATA ACCESSIBILITY

Effect size calculations: Dryad https://doi.org/10.5061/dryad.jm8ps6b.
